# Modulation of Lymphocyte Functions in the Microenvironment by Tumor Oncogenic Pathways

**DOI:** 10.3389/fimmu.2022.883639

**Published:** 2022-05-19

**Authors:** Barbara Seliger, Chiara Massa

**Affiliations:** ^1^ Institute of Medical Immunology, Martin Luther University Halle-Wittenberg, Halle (Saale), Germany; ^2^ Fraunhofer Institute for Cell Therapy and Immunology, Leipzig, Germany

**Keywords:** tumor infiltrating lymphocytes, tumor, oncogenic pathways, tumor suppressor genes, immunotherapy

## Abstract

Despite the broad application of different immunotherapeutic strategies for the treatment of solid as well as hematopoietic cancers, the efficacy of these therapies is still limited, with only a minority of patients having a long-term benefit resulting in an improved survival rate. In order to increase the response rates of patients to the currently available immunotherapies, a better understanding of the molecular mechanisms underlying the intrinsic and/or extrinsic resistance to treatment is required. There exist increasing evidences that activation of different oncogenic pathways as well as inactivation of tumor suppressor genes (TSG) in tumor cells inhibit the immune cell recognition and influegnce the composition of the tumor microenvironment (TME), thus leading to an impaired anti-tumoral immune response. A deeper understanding of the link between the tumor milieu and genomic alterations of TSGs and oncogenes is indispensable for the optimization of immunotherapies and to predict the patients’ response to these treatments. This review summarizes the role of different cancer-related, oncogene- and TSG-controlled pathways in the context of anti-tumoral immunity and response to different immunotherapies.

## Introduction

Malignant transformation is driven by the activation of oncogenes or inactivation of tumor suppressor genes (TSGs) leading to an enhanced and uncontrolled cell proliferation and survival. In addition to such cell-intrinsic effects, alterations in these pathways have also paracrine effects on the surrounding tumor microenvironment (TME), influencing also the frequencies and spatial distribution of immune cells (1, 2). Recently, evaluation of The Cancer Genome Atlas (TCGA) databases for the status of TSGs or oncogenes has highlighted multiple correlations with the amount and type of immune cell infiltrate as well as with the responsiveness or resistance to (immuno)therapies (3, 4).

We will start giving a general overview of how T lymphocyte responses are induced and controlled, about the tumor infiltrating immune cell repertoire and the intratumoral heterogeneity. Then, we will describe the role of selected oncogenes and TSGs and their associated pathways in modulating anti-tumor immune responses by affecting immune modulatory molecules in tumors and by inducing a tumor promoting and/or an immune suppressive TME. Moreover, we will discuss possible strategies to revert these processes in order to increase the clinical outcome of patients and enhance (immuno)therapeutic efficacy.

## Characteristics of T cell Activation and Response

In order to effectively and qualitatively eliminate pathogens as well as tumor cells, the adaptive immune system is relying on complex cell communication interactions between T lymphocytes and antigen presenting cells (APCs) such as dendritic cells (DCs) (5). At the level of whole cells, these interactions take place through the formation of the immunological synapse, a supra-molecular activation cluster (SMAC) including the T cell receptor (TCR) complex and multiple adhesion molecules that allow active signaling *via* the TCR. Thus, the local membrane topology has a large impact on TCR signaling (6, 7), which is a dynamic process generating a unique specificity and sensitivity of the T cell response (8). It is well known that T cell activation requires at least two initial signals: the so-called first signal, corresponding to the interaction of the TCR with its antigenic peptide presented by the major histocompatibility complex (MHC) antigen, while the second co-stimulatory signal is mainly provided by B7 ligands on APCs binding to the CD28 co-receptor of T cells (9). This leads to the formation of protein signaling complexes and subsequently to the activation of downstream pathways that induce the expression of interleukin (IL)-2 and other cytokines known to promote the expansion as well as the proliferation of T cells (10). Furthermore, T cell activation could be modulated by a series of spatial interaction processes, which allow biological decision between activation, anergy, apoptosis or exhaustion of T cells. Stimulation with only the first or the second signal, respectively, causes T cell anergy or apoptosis. In contrast, properly activated T cells are able to eliminate pathogen-infected cells as well as cancer cells, while avoiding damage to the healthy tissues of the host organism. The speed, sensitivity and specificity of this process is remarkable and conveyed by the activation of downstream pathways that regulate the expression and function of a plethora of immune modulatory genes/proteins (11). These include an upregulation of inhibitory molecules, a decrease of effector functions and a reduced proliferation that are required to shut-down the immune response after removal of the “unhealthy cells” (12). Furthermore, TCR signaling could regulate the stability and/or translation of cytokine mRNAs suggesting both a transcriptional as well as a post-transcriptional control.

## Important Features of Immune Checkpoints

Immune checkpoints (ICPs) are co-regulatory molecules controlling T cell activation and can be classified into stimulatory and inhibitory receptors. The former include CD28, CD27, ICOS, CD226, HVEM and OX40, while the latter comprise CTLA-4 (cytotoxic T lymphocyte-associated protein-4), PD1 (programmed cell death-1), TIGIT (T cell immunoreceptor with immunoglobulin and ITIM domain), VISTA (V-domain Ig suppressor of T cell activation) and LAG-3 (lymphocyte activation gene 3) (13–15). Some molecules, like BTLA and TIM-3 (T cell immunoglobulin and mucin domain 3), could exert both stimulatory as well as inhibitory activities depending on the cellular context (16–18). The stimulatory receptors are constitutively expressed or induced shortly after successful T cell activation, while the inhibitory ones are typically induced upon T cell stimulation as a negative feedback mechanism to avoid hyper-stimulation as well as to preserve healthy tissue integrity (19). Thus, a balance between co-stimulatory and co-inhibitory signals is required for the control of T cell responses and to ensure that activation is sufficient to eliminate pathogens and cancer cells, but not excessive since it would otherwise cause collateral damage (20). Due to these properties, T cell activation is tightly regulated and its inhibition is the key to prevent autoimmunity. This is in accordance with the function of immune checkpoint inhibitors (ICPis), which are able to enhance T cell anti-tumoral immunity, but can also induce autoimmune responses (21). Currently, monoclonal antibodies (mAbs) targeting the ICPs CTLA-4, PD1 and programmed death ligand 1 (PD-L1) have been approved by the Federal Drug Administration (FDA) and the European Medical Agency (EMA) for the treatment of diverse cancers including metastatic melanoma, non-small cell lung carcinoma (NSCLC), colorectal carcinoma (CRC), renal cell carcinoma (RCC) as well as head and neck squamous cell carcinoma (HNSCC) (22). Other ICPi are being evaluated for efficacy in multiple clinical trials. Mechanistically, ICPi could either compete for ligands of the activating co-receptors or control the surface expression of immune checkpoint receptors (ICP-Rs). Moreover, ICPi can interfere with the spatial arrangement necessary for efficient TCR signaling and thus recruit inhibitors of TCR activation, such as phosphatases, which can revert the TCR activation-induced phosphorylation and can induce diverse resistance mechanisms characterized by e.g. alterations of the interferon (IFN) pathway and of components of the antigen processing machinery (APM) (22, 23). Frequently, the efficacy of ICPi treatment is correlated to the tumor mutational burden (TMB) and to its immune contexture (24–27). Tumors with a high TMB are characterized by higher levels of neoantigens leading to an increased immune cell infiltration and display a favorable outcome and better responses to ICPi (28). For example, in triple negative breast cancer (TNBC), the TMB and the immune gene expression profile add an independent value for the prediction of pathologic complete remission, which has also relevance for the design of individually tailored (immuno)therapies (29). Interestingly, recent data indicate that the mutation quality is more important than their quantity. This is reflected by the fact that not all mutations are equivalent regarding their immunologic impact. For example, frame-shift mutations affecting RNA splicing or insertion/deletion generally create more immunogenic neoantigens than common single nucleotide mutations (27).

## ICPs as Tumor Suppressors in Some Cancers

Recently, next to their role in promoting or inhibiting T cell-based immunity, a direct role as tumor suppressors has been suggested for some ICPs. For example, the expression of the costimulatory CD80 molecule on tumor cells could have a pro- and anti-oncogenic role (30). Also for PD1 signaling in tumor cells opposing effects have been found depending on the tumor type analyzed and the presence or absence of adaptive immune cells. In melanoma and hepatoma, PD1 promoted tumor growth *via* activation of the mTOR pathway (31, 32). In contrast, in other tumors, including for example NSCLC, tumor cell intrinsic PD1 plays an anti-tumor role (33), which is due to a PD1-mediated inhibition of the AKT and ERK 1/2 pathways and has been associated with an increased tumor cell apoptosis and altered T cell proliferation (33, 34). Similarly, a growth inhibition of CTLA-4-expressing tumor cells was also reported (35). A general role of ICPs as tumor suppressors within malignant cells is strengthened by the identification of a meta-gene expression signature composed of CD27, CEACAM1, CTLA-4, LRIG1, PD-L2 and GITR within a collection of tumor cell lines, which was also associated with a prolonged survival phenotypes in clinical specimens (36). Expression of these ICPs was also associated with the inhibition of different oncogenic pathways including the transforming growth factor (TGF)-β signaling, angiogenesis, epithelial mesenchymal transition (EMT), hypoxia and metabolic processes (36).

## Immune Cell Repertoire in the TME and its Clinical Relevance

It has been demonstrated that the frequency of tumor-infiltrating lymphocytes (TILs) could serve as prognostic and predictive biomarker, in particular in the context of T cell-based immunotherapies (37, 38). Indeed, with the exception of RCC, tumor patients treated with ICPis and/or cancer vaccines have an increased response to treatment and a prolonged survival if they have a pre-existing local CD8^+^ T cell infiltration of the tumor (39, 40). In CRC, an immune score based on the number/density of lymphocyte populations in the invasive tumor margin (TM) and in the tumor center (TC) was found to have a statistically significant prognostic value, comparable to those of TNM staging and grading (25, 41, 42). In various other solid tumors, like gastric, bladder and breast cancer, such immune score has also been suggested to be a predictive marker for disease recurrence and represents now the first standardized immune-associated tumor classification in the clinic (43, 44). Stratification of patients based on immune characteristics was further extended to include also immune modulatory molecules. For example, a prognostic score as suppressive index for HNSCC was established by combining strong predictors for the survival of these patients, such as the abundance, location and spatial pattern of TILs to other immune markers, like expression of the human leukocyte antigen (HLA) class I (45, 46). However, not only the quantity, but also the quality of TILs is an important factor for patients’ outcome. The quality of T cell responses has been assessed by the antigen binding to their cognate receptors as well as by the expansion of both peripheral and intra-tumoral T cells. The TCR specificity is directed against neo-antigens and mutation-induced changes of cancer cell properties and thus directly associated with response to immunotherapy (47). In this context, it is noteworthy that not an individual lymphocyte subset is responsible for the tumor immune control, but rather the localization, clustering, interplay and spatial distribution and co-stimulation of all lymphocyte subsets are influencing the successful induction of anti-tumor immune responses. Regarding the composition of the TME, one could distinguish between tumors (i) with total lack of T cells, (ii) tumors with a non-T cell inflamed TME, in which tumors possess a number of antigens thereby excluding the reduced antigenicity as a pre-dominant evasion mechanism and (iii) T cell inflamed tumor lesions, where T cells recognize a large number of antigens resulting in proper anti-tumor immune responses (48). Therefore, a robust individualized immune signature predicting prognosis is required to identify patients who might have a benefit from immunotherapy.

## Intratumoral Heterogeneity and Immune Response

Despite extensive advancements in (immuno)therapies have been achieved during the last decade, treatment of tumor patients frequently confers an improvement only for a limited time frame. It was hypothesized that tumors with a complex heterogeneity might lead to a reduced patient´s survival, since these might be more difficult to eradicate. Despite tumor heterogeneity is highly linked to genomic instability, other factors for diversity are non-genetic defects mediated by tumor responses to microenvironmental factors including immune cell infiltration, metabolites and cytokines (49). This intratumoral heterogeneity (ITH) is affecting the interactions between tumor and immune cells, as supported by different mouse models, and influences also the response to immunotherapy (50). Cutting edge “omics” technologies combined to bioinformatic strategies allowed to study the ITH in more detail (51). Computer modeling of tumor/immune cell interactions including spatial and functional effects demonstrated that an increased cellular heterogeneity was associated with immune suppressive expression patterns (52) leading to a better survival of patients with low ITH (53, 54). Recent findings suggest that the ITH is an essential genetic determinant of anti-tumor immune responses. Both the number of distinct clones forming the tumor and the degree of their genetic divergence influence tumor aggressiveness (55). Due to an increased antigenic variability, the relative expression of each neoantigen is lower in tumors with increased ITH, thereby diminishing the homing of TILs to their target cells.

## Oncogenic and TSG Pathways in Tumors Influence the Frequency and Activity of Immune Cells

As described above, the TME is known to play a critical role in regulating anti-tumor immunity. Recent advances in genomic and transcriptomic strategies have provided evidences that molecular alterations in specific intrinsic pathways of tumor cells, such as induction/activation of oncogenic pathways as well as inhibition/inactivation of TSG, are not only involved in directly influencing the malignant phenotype of the tumor cells by modulating controlled cell death, cell differentiation, migration and genetic stability. Next to these oncogenic and tumor promoting programs, unexpected activities of oncogenes and TSGs on the regulation of the immune and tumor cell metabolism, on immune surveillance and on the epigenetic landscape have emerged (56). These processes can shape *via* paracrine mechanisms the TME thereby regulating the degree and functional status of infiltrating immune cells, which impacts the interaction between tumor cells and the host immune system and thus the general anti-tumoral responses. Indeed, several microenvironmental factors, e.g. the number of infiltrating immune cells, like macrophages, DC and neutrophils, as well as stromal cells, were significantly reduced in tumor lesions with mutated TSGs (4), while TSG non-mutated tumors might have an inflamed phenotype and thus be more likely to respond to ICPi therapies (4, 57). In addition, the expression of genes involved in lymphocyte differentiation as well as in interleukin production were downregulated (4). Based on these results, an increased understanding of the link between TME and oncogenic signaling is indispensable to get in depth insights into the role of oncogenes and/or TSGs in cancer immunity, which might also help to predict the patients’ response to (immuno)therapy. Recent work by Martin and co-authors based on a CRISPR screening approach demonstrated a low overlap between common TSGs in human cancers of different origin suggesting a tissue context-dependent role of TSGs in immune escape (2).

**Figure 1:**
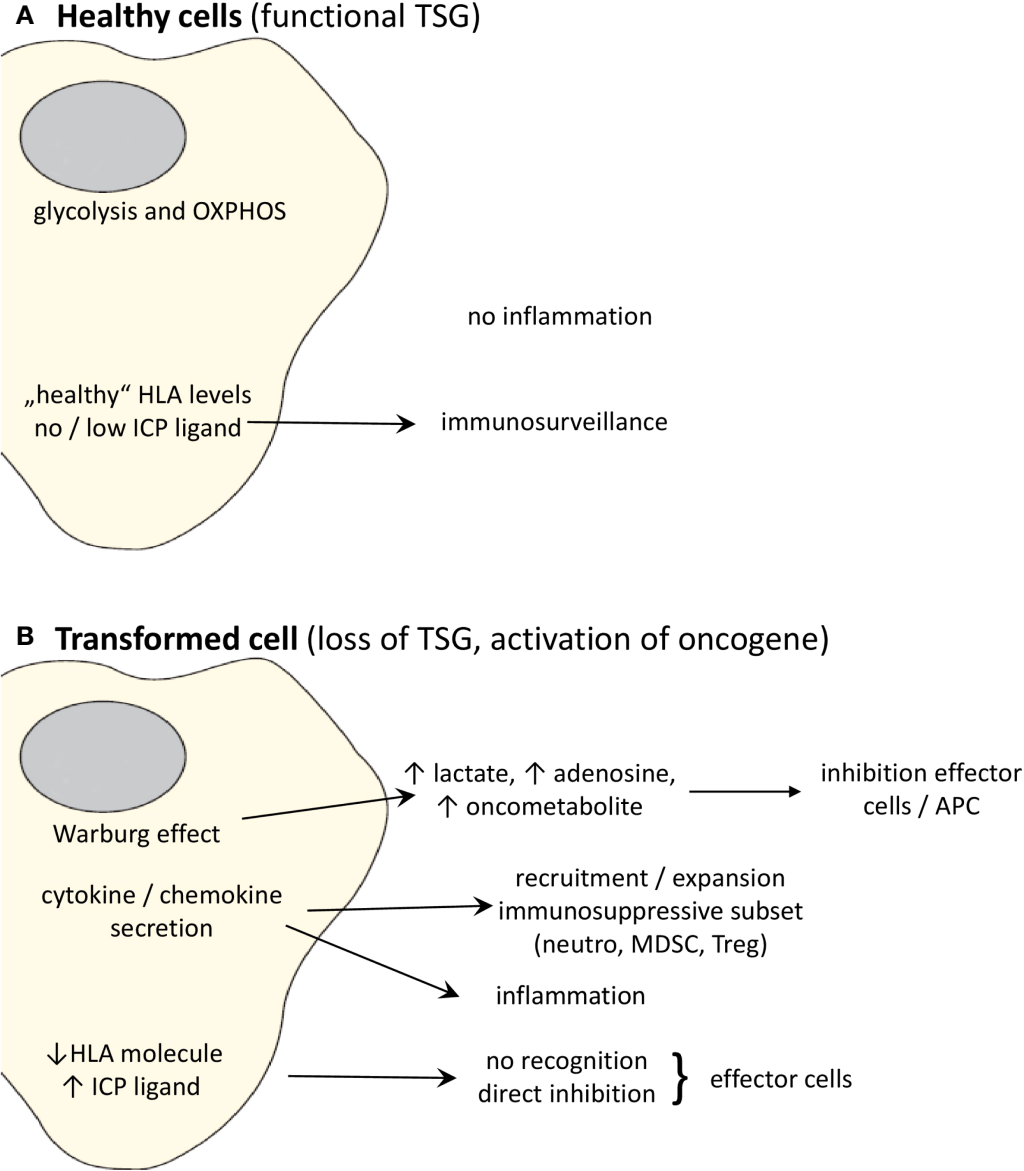
Effects of oncogenic activation and inactivation of TSG on the immune system. **(A)** Whereas “healthy” cells expressing non-mutated, functional TSG can be recognized by the immune system, **(B)** transformation to malignant cells due to activation of oncogenes and/or loss of TSGs changes the cell metabolism and cytokine/chemokine secretion pattern leading to the promotion of an immunosuppressive TME. Moreover, the altered oncogene and TSG expression causes a downregulation or loss of the expression of HLA class I molecules and upregulation of ligands for ICP leading to a direct escape from recognition by effector cells.

In the next paragraphs more detailed information for representative TSGs and oncogenes will be given, not only regarding the correlation between the expression of these genes and the immune microenvironment, but in some cases also on the specific pathway(s)/mechanism(s) leading to immune escape, as summarized in **Table 1** and **Figure 1**.

**Table 1:** Summary of the tumor cell intrinsic and extrinsic immune escape mechanisms mediated by oncogenes and TSGs.

A. Tumor-intrinsic mechanisms			
Oncogene / TSG	Variation in tumor cells		Consequences on immune cells
HER, K-RAS, LKB1, myc, NF1-PIK3CA, VHL	Tumor cell intrinsic	Reduced APM and HLA expression	Reduced TCR stimulation
IDH, myc		Reduced expression of NKG2D ligand	Reduced activation of NK cells
HER, myc, Wnt		Enhanced expression of inhibitory ligands (e.g. PD-L1)	Inhibition of effector cells
LKB1, p53		Reduced sensing of internal damage	No STING and innate immune cell recruitment / activation
**B. Tumor-extrinsic mechanisms**			
**Oncogene / TSG**	**Variation in tumor cells**		**Consequences on immune cells**
IDH, VHL, Wnt	Tumor cell extrinsic	Altered metabolism	Secretion of suppressive metabolites Depletion of metabolite recquired by effector cells
HER, IDH, K-RAS, LKB1, p53, PTEN, TET, VHL, Wnt		Altered secretion of cytokine and chemokine	Recruitment of suppressive immune cells and tumor promoting cells over APC and effector cells. “Wrong” polarization of immune cells

Exemplary, in the case of NSCLC, the presence of different driver mutations in addition to K-RAS mutation results in alteration in the immune infiltrate composition as well as in the tumor susceptibility and response to different immunotherapies. Tumors with a mutation in the TSG liver kinase B1 (LKB1) were found to be associated with a worse prognosis, reduced immune infiltration and PD-L1 expression and thus a lower response to ICPis than tumors with mutated TP53 (58). Experiments performed in murine models of K-RAS-driven NSCLCs highlighted that the additional presence of the LKB1 deletion induced an immunosuppressive status characterized by the expansion of neutrophils or myeloid-derived suppressor cells (MDSCs) and a reduced and impaired T cell infiltrate, characterized also by a decreased cytokine production and a more exhausted phenotype, as highlighted by the expression of different ICPs, like PD1, LAG-3 and TIM-3 (59, 60). Inhibition of IL-6 and thus of neutrophils in the first model was able to increase T cell infiltration of tumors, but did not enhance response to PD1 therapy (59). In contrast, depletion or functional inhibition of MDSC in the second setting synergized with anti-PD1 therapy, but only in a tumor model with high TMB (60). Loss of LKB1 also reduced the expression of components of the dsDNA sensing system, like the stimulator of interferon genes (STING). Since impaired LKB1 expression is associated with damaged mitochondria and thus release of DNA into the cytosol, reduced levels of STING avoid the induction of STAT1 signaling and production of chemokines like CXCL10 as well as expression of PD-L1 (61). Finally, a direct role of LKB1 in reducing T cell-mediated tumor cell recognition despite high TMB levels has been linked to the suppression of different APM components including various proteasomal subunits (62).

On its own, K-RAS has diverse immunological consequences. Mutated K-RAS expression is associated with an increased expression of PD-L1 (63) and a downregulation of HLA class I antigens and of APM components suggesting a link between K-RAS activation and control of immune recognition (64–66). Interestingly, this could be reverted *in vitro* by the treatment with IFN-γ or with inhibitors of the MAPK or of the K-RAS G12C mutation (67, 68). Furthermore, in different models of K-RAS activation including CRC, oncogenic K-RAS represses the expression of interferon-regulatory factor (IRF)2, which directly affects CXCL3 expression thereby promoting the influx of MDSC into the TME (69, 70). A global downregulation of immune cells was detected in lung adenocarcinoma patients harboring mutations in the K-RAS G12C gene, which correlates with the presence of downregulated transcripts (71).

The WNT-β-catenin pathway plays many roles within tumor cells to foster their malignant transformation and to keep the cancer stem cell properties. Recently, it has also been identified as one of the important oncogenic pathways playing a direct role in the immune evasion through different mechanisms. For example, it influences the tumor metabolism by inducing the Warburg effect (72) and upregulates the ICPs CTLA-4 (73) and PD-L1, either directly (74, 75) or indirectly *via* its target myc (76). Moreover, in different tumor types, such as melanoma, bladder cancer, CRC as well as HNSCC an inverse correlation between WNT-β-catenin activation and T cell infiltration was found (77). Decreased secretion of immune cell attracting chemokines leading to impaired recruitment of DCs into the TME (78) was responsible for such an impaired T cell recruitment as well as priming (78). The expression of WNT-β-catenin was also associated with the infiltration of regulatory T cells (Tregs), their survival and activity as well as with the modulation of the innate immunity (79). Moreover, WNT can promote the expression of CD73 (80) thus enhancing the levels of extracellular adenosine that can further impair T cell functions (81, 82). The WNT-mediated immune escape was linked to the resistance to ICPi therapy suggesting that WNT activation is a potential biomarker for patients’ stratification for therapy (75). Due to the many roles of this pathway, its direct therapeutic targeting is complex, but first approaches have recently been undertaken. Preliminary data confirmed that treatment with WNT inhibitors were able to revert the immune suppressive conditions (83, 84) and could also enhance response to different immunotherapeutic approaches ranging from adoptive transfer to ICPi. The tumor rejection was correlated both to changes in the tumor cells, like an upregulation of MHC class I surface expression (85) and modulation of PD-L1 expression (86) that render tumor cells more sensitive to cytotoxic T lymphocytes (CTLs) as well as to alterations in the TME, which reverted the immune suppressive conditions and allowed recruitment of effector cells (87, 88).

Increased transcription rates of the myc oncogene due to gene amplification or constitutive overexpression not only affects intrinsic properties of tumors, like increased proliferation and survival, but also their immunogenicity. Indeed, myc can downregulate HLA class I antigen expression, while inducing ICPs, like e.g. PD-L1 (63, 76) and CD47 (76) thereby influencing the repertoire of infiltrating immune cell (76, 89–91). In acute myeloid leukemia (AML) myc overexpression is accompanied by an immature myeloid differentiation due to epigenetic regulation of cell death and differentiation (92). Furthermore, myc overexpression is associated with early disease progression from myelodysplastic syndromes to AML (93). For these reasons, different treatment modalities targeting the myc/CD47 axis are tested for therapeutic usage (94, 95). Myc has also been involved in the evasion from natural killer (NK) cell surveillance by reducing the expression of ligands for the NKG2D activating receptor (96). Moreover, myc alone or in cooperation with RAS, affects the expression of chemokines leading to a more immune suppressive infiltrate (70, 97). In contrast, in gastric adenocarcinoma high myc expression levels are a good prognostic factor associated with low numbers of Tregs and low expression levels of PD-L1 (98).

Oncogenic signaling mediated by members of the HER gene family, in particular by EGF-R/HER-1 and HER-2/neu, results in an upregulation of PD-L1 expression in various cancer types, including HNSCC and NSCLC (99–101). This is mediated by an increased JAK2 and STAT3 expression and is accompanied by secretion of proinflammatory cytokines (102). Furthermore, activation of HER-1 and HER-2/neu is inversely associated with the expression of HLA class I antigens and APM components and prevents CTL-mediated immune recognition (103, 104). HER-2/EGF-R overexpressing cells secrete high levels of the immune suppressive cytokines TGF-β, IL-10 and vascular endothelial growth factor (VEGF), which affects the phenotype and function of TILs. Moreover, EGF-R mutated tumors can further suppress immune responses by an enhanced expression of CD73 and thus increased levels of adenosine in the TME (105, 106). Thus, a deregulated oncogenic growth factor signaling is linked with an inflammatory pro-tumorigenic and immune inhibitory TME.

Next to oncogenic activation, loss of TSGs have been shown to play a role in immune evasion (1). An immune regulatory role for the phosphatase and tensin homologue deleted on chromosome 10 (PTEN) was demonstrated. Evaluation of TCGA datasets including different cancer types positively correlated the expression of PTEN with the amount of T cell infiltrate (107) and inversely with the frequency of Tregs (108). Mechanistically, prostate cells that have lost PTEN secrete high levels of CSF and IL-1β, resulting in the recruitment and expansion of MDSC that inhibit T cell infiltration as well as their functions (109). Moreover, in different tumors, the presence of a PTEN mutation correlated with a missing response to PD1 blockade (110–113). PTEN reactivation in a preclinical model has been shown to enhance anti-tumor immunity (114). In contrast, evaluation of endometrial carcinoma associated the loss of PTEN with favorable prognosis (115).

Regarding p53, the story is even more complex, since mutations not only lead to the loss of the suppressive properties associated with a deregulated cell proliferation, but some of them provide also “gain of function” resulting in an oncogenic activity of p53 that influences the interaction with cells from the microenvironment including hematopoietic cells and stromal components (116, 117). As a consequence, the expression of inflammatory mediators as well as of chemokines is affected, resulting in a distinct immune infiltrate composition with more M2 macrophages and less T cells (118). In AML, a higher T infiltrate was found in mutated patients, but this exhibited a more exhausted phenotype (119). In lung cancer, a p53-relevant gene signature was associated with an altered immune infiltration and clinical outcome resulting in its establishment as a prognostic biomarker (120). Mechanistically, it has been demonstrated that mutp53 promotes an inflammatory, pro-tumoral TME by either promoting IL-1β secretion (121) or by enhancing myeloid cell recruitment *via* CCL2 and tumor necrosis factor (TNF)-α (122). In addition, the mutant p53-mediated alterations of the TME include a pro-invasive extracellular matrix structure, with enhanced cancer-associated fibroblast activity disabling innate immune responses (116). Indeed, p53 can promote tumor survival by suppressing the activation of the innate sensing pathway of TKB1/STING thereby saving cells from apoptosis and inhibiting activation and recruitment of effector cells, like NK cells and CD8^+^ T lymphocytes (123). In this context, it is also noteworthy that TP53 has been shown to increase MHC class I expression by upregulation of APM components, such as the endoplasmic reticulum resident aminopeptidase ERAP1 (124). In SCLC, a dual inactivation of TP53 and RB was found, which resulted in a global chromosomal instability. This was accompanied by a high incidence or loss of immune genes, including components of the IFN-γ and HLA class I pathway (125). Thus, the inactivation of p53 has an impact on the immune and inflammatory hallmarks of cancer. Therapeutically, a recently described antibody recognizing a peptide encompassing the most common mutation of p53 in association with HLA-A02 has been transformed into a bispecific antibody that has been tested in preclinical *in vivo* models for its ability to retarget effector cells to tumor cells carrying the mutation (126).

Next to the activation/inactivation of these prominent oncogenes/TSGs, additional pathways have been shown to affect anti-tumor immunity, which are summarized below.

The ten-eleven translocation (TET) family of proteins is frequently mutated in hematopoietic malignancies (127), whereas in solid tumors a reduced activity of these enzymes highlighted by reduced presence of their metabolite 5hmC is more frequently found (128). Targeted deletion of the *TET2* gene within murine melanoma cells highlighted also consequences on the immune system with a reduced T cell infiltration of the mutated tumor. This was linked to an altered signaling of the IFN/JAK pathway and reduced production of chemokines (129). In the opposite direction, the activation status of *TET2* within immune cells has consequences on the progression of solid tumors, but, due to the role of TET in epigenetic regulation, the consequences are highly context-dependent. Indeed, in a melanoma setting removal of *TET2* from myeloid cells reverted their immune suppressive phenotype induced by IL-1 and allowed a type 1 polarization leading to the recruitment of T cells that could contrast tumor growth (130). In contrast, in lung cancer the TET2 knockout in myeloid cells promoted angiogenesis and tumor progression *via* a S100A8/A9 - VEGFα loop (131). Similarly, using hepatoma as well as breast cancer cell lines, a faster tumor growth in TET2 knockout mice was found due to an IL-6-mediated expansion of MDSCs and a consequently reduced T cell infiltration (132).

Isocitrate dehydrogenase (IDH)-1 and -2 play an important role in stratifying glioma patients. Indeed, tumors carrying IDH-1/IDH-2 mutation(s) have lower levels of PD-L1 and also a reduced T cell infiltration despite an increased patients’ survival (133). This is partly due to a reduced production of attracting chemokines (134) and to a modulation of the suppressive myeloid infiltrate (135). Mechanistically, the mutated forms alter tumor cell metabolism and epigenetic patterns, but also acquire the capability to produce D-2-hydroxyglutarate, that is an oncometabolite able to inhibit T cell functions (136, 137), and to escape from NK surveillance by reducing the expression of the NKG2D ligand (138). Moreover, mutated tumors produce more extracellular vesicles that can promote an immune suppressive milieu (139, 140). Therapeutically, different inhibitors are currently being investigated (141). Since one IDH-1 mutation has been shown to generate a neo-antigen that is recognized by patients’ CD4^+^ T cells (142), vaccination trials targeting this mutation are ongoing (NCT02454634, NCT02193347 and NCT02771301) and first results on safety have been recently reported (143).

Inactivation of the von Hippel Lindau (VHL) gene has been frequently demonstrated as a driving factor in RCC, particularly in RCC of the clear cell subtype. Upon its mutation, the hypoxia inducible factor (HIF)-1α and -2α are not undergoing correct degradation leading to alterations of their regulated pathways, namely metabolism, proliferation and angiogenesis with consequences also on immune system cells. Indeed, it is known that the enhanced use of anaerobic glycolysis due to the enhanced activation of HIF results in glucose depletion as well as hypoxia and enhanced lactate secretion (144), all factors having negative consequences on the functional capabilities of effector cells. VHL inactivation results also in changes in the tumor secretomes that can affect T cells functions, for example due to enhanced levels of the MnSOD2 enzyme that cause redox stress in T cells thereby impairing their functionality (145). Moreover, the enhanced secretion of VEGF that mediates angiogenesis is also involved in the expansion and recruitment of immune suppressive MDSC (146) that will further suppress effector cells. In addition, comparison of specimens with or without VHL mutations has highlighted differences in the tumor immune signatures that can also associate with response to therapy (147, 148). Tumors with loss of VHL function due to mutations in the *VHL* gene or *via* its epigenetic control displayed lower levels of Treg and higher frequencies of NK cells that were also more cytotoxic in *in vitro* assays (149). Interestingly, mutated VHL reduced the expression of HLA class I antigens that might protect tumor cells from T cell recognition, but renders them more susceptible to NK cell cytotoxicity (150). *Via* HIF, VHL inactivation can induce the expression of CD70 on RCC specimens resulting in an enhanced infiltration of CD27^+^ T cells accompanied by a worse patients’ outcome (151). The VHL status is also modulating the expression of PD-L1 due to its effects on HIF2α levels (152–154).

In glioblastoma, mutations in NF1 and PIK3CA have been shown to modulate the interferon-regulatory factor (IRF)-1, which activates the expression of downstream target genes including APM components, which are associated with an increased lymphocyte infiltration and a worse survival of patients (155).

The guanine nucleotide binding protein α13 (Gna13) also displays immune regulatory activities *via* its action on the TME, since it inhibits the expression of CCL2. Indeed, Gna13 loss in different murine models resulted in an increased secretion of CCL2 leading to enhanced recruitment of tumor associated macrophages (TAMs) (2).

Many of the indicated oncogenes/TSGs signal through the nuclear factor kappa B (NF-ĸB), which is involved in promoting tumorigenesis and inflammation-induced carcinogenesis by locally inhibiting innate and adaptive host immune responses (156). Activation of NF-ĸB signaling has been associated with the control of oncogenic functions and tumor progression as well as with an increased inflammation through its function in innate immune cells (157, 158). Furthermore, activation of NF-_K_B has also been demonstrated to promote resistance to programmed cell death (159).

## Conclusion

Many progresses have been made and correlations found between the genetic drivers of tumor transformation and the shaping of the TME leading to a better understanding of the mechanisms involved in the resistance or responsiveness to (immuno)therapy. For this reason, a number of approaches are currently being tested to recover the function of TSGs or to disrupt the regulatory axes involved in oncogenic activation in order to reduce the immune suppressive function and enhance immunity. Initial results in different experimental models appear to be promising, with restoration of functional TSGs resulting in the modulation of the TME and an improved activity of ICPis (114, 116). However, due to the partially opposing roles of these pathways on the immune system in different tumor types, the path to effective personalized medicine is still long.

## Author Contributions 

All authors listed have made a substantial, direct, and intellectual contribution to the work and approved it for publication.

## Funding

This work was supported by a grant from the Deutsche Krebshilfe 70113311 (BS) and the Deutsche Forschungsgemeinschaft (DFG, SE 581/33-1). We acknowledge the financial support of the Open Access Publication Fund of the Martin-Luther-University Halle-Wittenberg.

## Conflict of Interest

The authors declare that the research was conducted in the absence of any commercial or financial relationships that could be construed as a potential conflict of interest.

## Publisher’s Note

All claims expressed in this article are solely those of the authors and do not necessarily represent those of their affiliated organizations, or those of the publisher, the editors and the reviewers. Any product that may be evaluated in this article, or claim that may be made by its manufacturer, is not guaranteed or endorsed by the publisher.

## Abbreviations

AML, acute myeloid leukemia; APC, antigen presenting cell; APM, antigen processing machinery; CRC, colorectal carcinoma; CTL, cytotoxic T lymphocyte; CTLA-4, cytotoxic T lymphocyte-associated protein-4; DC, dendritic cell; EMA, European Medical Agency; EMT, epithelial mesenchymal transition; Gna, guanine nucleotide binding protein; HIF, hypoxia inducible factor; FDA, Federal Drug Administration; HLA, human leukocyte antigen; HNSCC, head and neck squamous cell carcinoma; ICP, immune checkpoint; ICPi, immune checkpoint inhibitor; ICP-R, immune checkpoint receptor; IDH, isocitrate dehydrogenase; IFN, interferon; IL, interleukin; IRF, interferon-regulatory factor; ITH, intratumoral heterogeneity; LAG-3, lymphocyte activation gene 3; LKB1, liver kinase B1; mAb, monoclonal antibody; MAPK, mitogen-activated protein kinase; MDSC, myeloid-derived suppressor cell; MHC, major histocompatibility complex; NF-ĸB, nuclear factor kappa B; NK, natural killer; NSCLC, non-small cell lung carcinoma; PD1, programmed cell death-1; PD-L1, programmed death ligand 1; PFS, progression-free survival; PI3K, phosphatidylinositol 3-kinase; PTEN, phosphatase and tensin homologue deleted on chromosome 10; RCC, renal cell carcinoma; SMAC, supra-molecular activation cluster; STING, stimulator of interferon genes; TAM, tumor-associated macrophages; TCGA, The Cancer Genome Atlas; TC, tumor center; TCR, T cell receptor; TET, ten-eleven translocation; TGF, transforming growth factor; TIGIT, T cell immunoreceptor with immunoglobulin and ITIM domain; TIL, tumor infiltrating lymphocyte; TIM-3, T cell immunoglobulin and mucin domain 3; TM, tumor margin; TMB, tumor mutational burden; TME, tumor microenvironment; TNBC, triple negative breast cancer; TNF, tumor necrosis factor; Treg, regulatory T cell; TSG, tumor suppressor gene; VEGF, vascular endothelial growth factor; VHL, Von Hippel Lindau; VISTA, V-domain Ig suppressor of T cell activation;

## Glossary

**Table:**

AML	acute myeloid leukemia
APC	antigen presenting cell
APM	antigen processing machinery
CRC	colorectal carcinoma
CTL	cytotoxic T lymphocyte
CTLA-4	cytotoxic T lymphocyte-associated protein-4
DC	dendritic cell
EMA	European Medical Agency
EMT	epithelial mesenchymal transition
Gna	guanine nucleotide binding protein
HIF	hypoxia inducible factor
FDA	Federal Drug Administration
HLA	human leukocyte antigen
HNSCC	head and neck squamous cell carcinoma
ICP	immune checkpoint
ICPi	immune checkpoint inhibitor
ICP-R	immune checkpoint receptor
IDH	isocitrate dehydrogenase
IFN	interferon
IL	interleukin
IRF	interferon-regulatory factor
ITH	intratumoral heterogeneity
LAG-3	lymphocyte activation gene 3
LKB1	liver kinase B1
mAb	monoclonal antibody
MAPK	mitogen-activated protein kinase
MDSC	myeloid-derived suppressor cell
MHC	major histocompatibility complex
NF-ĸB	nuclear factor kappa B
NK	natural killer
NSCLC	non-small cell lung carcinoma
PD1	programmed cell death-1
PD-L1	programmed death ligand 1
PFS	progression-free survival
PI3K	phosphatidylinositol 3-kinase
PTEN	phosphatase and tensin homologue deleted on chromosome 10
RCC	renal cell carcinoma
SMAC	supra-molecular activation cluster
STING	stimulator of interferon genes
TAM	tumor-associated macrophages
TCGA	The Cancer Genome Atlas
TC	tumor center
TCR	T cell receptor
TET	ten-eleven translocation
TGF	transforming growth factor
TIGIT	T cell immunoreceptor with immunoglobulin and ITIM domain
TIL	tumor infiltrating lymphocyte
TIM-3	T cell immunoglobulin and mucin domain 3
TM	tumor margin
TMB	tumor mutational burden
TME	tumor microenvironment
TNBC	triple negative breast cancer
TNF	tumor necrosis factor
Treg	regulatory T cell
TSG	tumor suppressor gene
VEGF	vascular endothelial growth factor
VHL	Von Hippel Lindau
VISTA	V-domain Ig suppressor of T cell activation
